# Extraction of a Foreign Body From the Middle Ear Following a Complicated Hearing Aid Fitting: A Case Report

**DOI:** 10.7759/cureus.36499

**Published:** 2023-03-22

**Authors:** Haya Alsubaie, Saif Alghamdi, Abdulaziz Almalki, Omar S Almansouri, Fahad A Alsulami, Sumayya A Bafail

**Affiliations:** 1 Otolaryngology, Head and Neck Surgery, King Saud Bin Abdulaziz University for Health Sciences College of Medicine, Jeddah, SAU; 2 Otolaryngology, Head and Neck Surgery, King Abdulaziz Medical City, Riyadh, SAU; 3 Otolaryngology, Head and Neck Surgery, King Fahad Military Medical Complex, Jeddah, SAU; 4 Medicine, King Saud Bin Abdulaziz University for Health Sciences College of Medicine, Jeddah, SAU; 5 Medicine and Surgery, King Saud Bin Abdulaziz University for Health Sciences College of Medicine, Jeddah, SAU

**Keywords:** foriegn body, bone anchored hearing aid, ear molding, implantation otology, foreign body removal, unusual foreign body

## Abstract

Ear molding is a safe way to evaluate the ear for hearing aid fitting. A very rare complication of ear molding is the entry of a foreign body into the middle ear. We report the case of a three-year-old boy who had a perforated left tympanic membrane and bilateral sensorineural hearing loss that required the use of hearing aids. During the ear molding procedure, the molding material was unintentionally introduced into his middle ear cavity, necessitating immediate surgery to remove it. Such patients with tympanic membrane perforation must be handled cautiously while an aural impression is taken via ear molding to prevent introducing a foreign body into the middle ear.

## Introduction

Ear molding is a common office procedure performed by audiologists or hearing aid specialists to obtain an aural impression for hearing aid fitting. The procedure is generally safe with rare complications if contraindications such as postoperative ear infections, active infections, and impacted wax are avoided [1]. Although tympanic membrane perforation is not a contraindication to ear mold fitting, care must be taken to guard against the possibility of introducing a foreign body (FB) to the middle ear through the perforated tympanic membrane while removing the material [1,2]. Immediate symptoms of ear pain, hearing loss, tinnitus, and vertigo can be expected if molding material penetrates the middle ear cavity [2]. Moreover, patients' symptoms, such as hearing loss and discharge, can manifest days after obtaining the aural impression [3]. Upon diagnosis of FB in the middle ear, prompt referral to an otolaryngologist should be done for possible surgical intervention [2,4]. We report a surgically managed case of an iatrogenic FB introduced to the middle ear cavity while obtaining an aural impression.

## Case presentation

**Figure 1:**
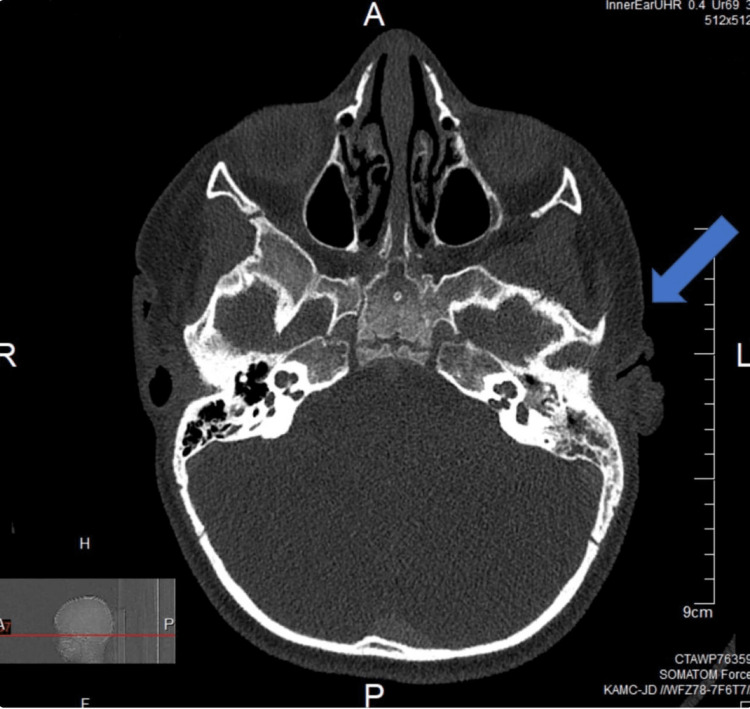
A preoperative CT scan of the mastoid at the level of the mesotympanum showing the opacification of the middle ear involving the sinus tympani, mastoid air cells, as well as the facial recess.

A three-year-old boy was referred to our tertiary care center with delayed speech development and hearing impairment since the age of one year. The patient had no significant medical history apart from congenital glaucoma, which had been managed surgically. The patient underwent cardiological, behavioral, and developmental screening that showed no abnormalities. An audiological assessment revealed a bilateral sensorineural hearing loss that required hearing aids. When the patient underwent an otolaryngology evaluation, the examination revealed a perforated left tympanic membrane and a right tympanic membrane with a pinpoint perforation. After the patient's referral to audiology for a hearing aid fitting, the patient was referred back to otolaryngology for an inadvertent molding material insertion into the middle ear during the hearing aid fitting. Computed tomography (CT) imaging following the complication showed the foreign body (FB) encasing the ossicles and extending into the Prussak pouch (Figure 1).

The patient underwent urgent surgery for the removal of the FB. Through a trans-canal microscopic approach, the FB was visualized and impacted the external canal, causing edema and perforation of the tympanic membrane and extending to middle ear structures including the sinus tympani, facial recess, and anterior epitympanic area. The FB was partially removed after multiple trials, saving the ossicles from any damage. After the inability to remove all of the remnants due to poor visualization of the FB, the patient underwent revision surgery after three weeks for removal of the remaining material of the FB and tympanoplasty of both ears. During the revision surgery, the tympanomeatal flap was elevated, and the examination of the middle ear showed part of the FB in the attic and the posterior mesotympanum. The long process of the incus was found to be eroded along with the incudostapedial joint. The short process, the body of the incus, and part of the malleus were also found to be eroded by the inflammatory process of the FB. A trans-canal endoscopic atticotomy was done during the revision surgery. For full visualization of the FB, the short part of the long process of the incus was sacrificed, after which complete removal of the FB was made along with tympanoplasty of the left and right ears.

## Discussion

Ear molding is generally safe, with rare complications [1]. But even if contraindications such as postoperative ear, active infections, and impacted wax are avoided, complications like a foreign body in the middle ear cavity or ear canal, traumatic tympanic membrane perforation, and ossicular chain disruption can occur [1,5]. In those high-risk patients, it is imperative to suspect the introduction of impression material into the middle ear cavity if patients complain of acute pain, hearing loss, or acute vestibular symptoms [6,7]. Moreover, high-risk patients can also have late presentations with chronic ear discharge and hearing loss [8]. In the case of complicated ear mold fittings, blind removal of the mold material should be avoided to avoid additional damage. A CT of the temporal bone is advised in such cases if the tympanic membrane cannot be visualized or middle ear involvement is suspected [2]. Cases of foreign bodies from impression material during ear canal and middle ear fittings are underreported and likely much more common than the literature suggests [5]. Impression-material foreign bodies can also cause serious complications, mostly related to long-term hearing loss [5]. As a result, clinical guidelines and approaches have emerged to facilitate better outcomes and prevent complications. Cho et al. proposed a clinical guideline algorithm in which they highlighted the need for a prompt CT temporal scan when middle-ear cavity involvement is clinically suspected [5]. Cho et al. have suggested doing a temporal CT scan upon the presence of acute symptoms during impression taking or if they are considered high-risk patients [5]. They defined high-risk patients as those with tympanic-membrane perforations, chronic suppurative otitis media, a narrow external auditory canal, a history of tympanomastoidectomy, or a history of tube insertion [5]. Their proposed surgical approach in the case of confirmed middle ear cavity involvement is tympanomastoidectomy [5].

Our presented case highlights some of the consequences the patient might experience due to the impression material. Initial partial removal of the FB through a trans-canal approach was uneventful but also unsuccessful because of the deep involvement of the impression material. Revision surgery through a tympanomeatal flap was needed for the full removal of the material, followed by tympanoplasty. As a result of deep involvement with the material, the short part of the long process of incus was sacrificed for full visualization of the FB. Another direct damage caused by the impression was the erosion of the long process of the incus along with the incudostapedial joint. For the prevention of these and other complications, utmost care must be taken when applying an ear mold, and careful examination of the ears for high-risk features is advised.

## Conclusions

We present the case of a three-year-old boy with a history of tympanic perforation referred to our clinic for left-middle ear FB as a complication of the ear mold fitting. The patient underwent a temporal bone CT scan prior to surgery. Full removal of the FB was successful after revision surgery through a trans-canal atticotomy. The introduction of impression material into the middle ear cavity is a possible complication, especially in patients with tympanic membrane perforations. Prompt diagnosis via CT imaging upon clinical suspicion of middle ear FB can aid in faster management and the prevention of further complications.
